# Spontaneous Right Thalamic Hemorrhage With Intraventricular Extension in a Two-Month-Old Preterm Infant Initially Suspected of Having Cow’s Milk Protein Allergy

**DOI:** 10.7759/cureus.102493

**Published:** 2026-01-28

**Authors:** Tuqa A Abdulsalam, Rehab Musa, Mohammed Aldirawi, Moataz Hamdi, Lemis Yavuz

**Affiliations:** 1 General Pediatrics, Al Jalila Children's Specialty Hospital, Dubai, ARE

**Keywords:** colic, cow's milk protein allergy, infantile colic, intraventricular hemorrhage (ivh), late preterm neonates, pediatric intracranial hemorrhage, platelet function disorder, spontaneous intracerebral hemorrhage, thalamic hemorrhage

## Abstract

Nontraumatic spontaneous intracerebral hemorrhage in infants is rare and usually occurs due to infection, trauma, or known coagulopathy, most often originating from the thalamus and extending into the ventricle. In this case report, we describe a two-month-old late preterm female infant (36 weeks of gestational age) who was brought in due to irritability, projectile vomiting, and feeding difficulties. The clinical history suggested cow's milk protein allergy, as the baby had recently been switched to formula. The neurological signs observed during physical examination, including upward eye deviation and lack of visual tracking, prompted neuroimaging, which revealed a right thalamic hemorrhagic infarct with intraventricular hemorrhage. The patient was stabilized and transferred to the local tertiary neurosurgical center. Workup for thrombophilia and vascular malformations was initiated. The child recovered uneventfully and demonstrated normal developmental status at follow-up.

## Introduction

Intracerebral hemorrhage (ICH) in infants and young children is rare, with an annual incidence of one to five per 100,000 children [1]. Among neonates, particularly those born prematurely, hemorrhage most commonly occurs in the germinal matrix with intraventricular extension because of the fragile nature of cerebral vascularity and changing cerebral perfusion pressures [2]. Spontaneous parenchymal hemorrhages in nonneonatal infants, including those related to deep gray matter compartments such as the thalamus, however, are rare and may be triggered by clinical suspicion of associated vascular or hematologic disorders [3].

The thalamus, with its rich blood supply from small penetrating arteries, is a common site for hypertensive hemorrhage in adults. Nevertheless, this is an unusual form of bleeding for infants of this age group. In the pediatric population, thalamic hemorrhage is mainly caused by arteriovenous malformations (AVMs), cerebral venous sinus thrombosis (CVST), and coagulopathies (e.g., factor deficiencies, thrombophilia), as well as traumatic head injury [4]. However, when presenting spontaneously and in the absence of trauma or infection, they merit a complete work-up to exclude underlying systemic or structural etiologies.

Intraventricular hemorrhage (IVH) is a frequent occurrence in the most premature neonates as a complication of germinal matrix hemorrhage. Still, it is rare for late-preterm and term infants not to be complicated by these risks [5]. When it occurs in older infants, IVH is commonly accompanied by parenchymal hemorrhage, as in our case, or from ruptured vascular malformations. Ventricular seepage has been observed to result in obstructive hydrocephalus, increased intracranial pressure, and chronic neurodevelopmental impairment if not managed appropriately [4].

We report a rare case of spontaneous right thalamic hemorrhage with intraventricular extension in a two-month-old preterm infant. The child initially exhibited gastrointestinal and eye symptoms before neuroimaging revealed a right thalamic hemorrhage. This case emphasizes the value of early neuroimaging and a multidisciplinary approach in atypical infant cases and demonstrates a favorable outcome with conservative management.

## Case presentation

A two-month-old female infant, delivered at 36 weeks, presented to the emergency department with a one-day history of continuous vomiting, poor feeding, irritability, and reduced visual interest. She was diagnosed with a case of cow's milk protein allergy (CMPA) at one week of age and was started on an amino acid formula, which she had tolerated well. One week before this presentation, a decision was made to alter her milk to an extensively hydrolyzed formula on medical advice for a presumed diagnosis of colic. On the eighth day of the new formula, the patient developed large-volume, nonbilious, nonbloody emesis, watery yellow-green stools, and increasing irritability. Given a suspicion of the return of CMPA symptoms, her mother switched back to the initial amino acid formula. Still, vomiting and irritability continued, and she also developed worrying neurological features like persistent upgaze and loss of visual following.

There was no history of fever, seizure, trauma, cyanosis, respiratory distress, or any abnormal movement. Developmental history was age-appropriate until the onset of symptoms (follows a social smile and visual tracking). Family history was significant for a maternal aunt who had a history of venous thromboembolism at 29 years of age and was on long-term anticoagulation. No consanguinity was reported, and there was no family history of congenital abnormalities, stroke, or coagulopathy.

The baby was irritable and had periods of high-pitched crying that could not be consoled. She was febrile and hemodynamically stable, with a capillary refill time of less than two seconds and oxygen saturation of 98% on room air. The anterior fontanelle was full but did not bulge. She exhibited poor visual fixation with sustained upward gaze, but no focal motor deficits were observed neurologically. The abdominal examination was soft and nontender. Pediatric surgery was consulted for vomiting and suspected abdominal pathology; abdominal radiograph and ultrasound were unremarkable. As there were reduced movements of the left lower limb and tenderness upon palpation of the left hip, an ultrasound of the hip was performed. However, no evidence of effusion or dislocation was detected due to the presence of neurologic signs, gaze fixation, and unresponsiveness. Therefore, an intracranial ultrasound was requested in the emergency department.

**Figure 1:**
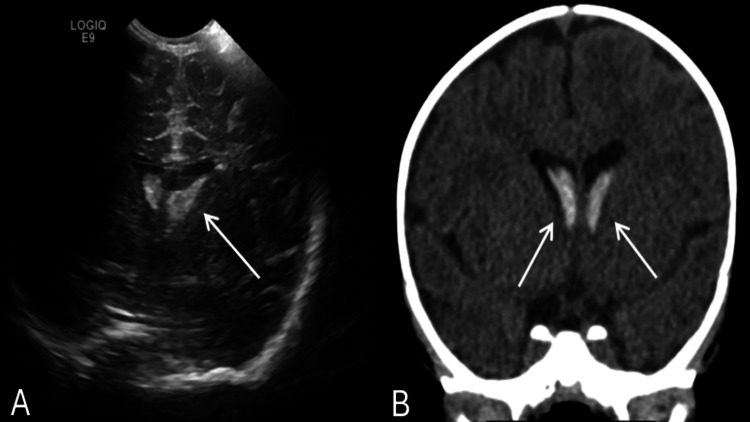
Neuroimaging findings of spontaneous right thalamic hemorrhage with intraventricular extension (A) Cranial ultrasound demonstrating a hyperechoic lesion within the right thalamus measuring 2.7 × 2.5 × 2.4 cm with echogenic extension into the lateral ventricles, suggestive of a hemorrhagic infarct and germinal matrix hemorrhage. (B) Noncontrast CT of the brain showing a hypodense, swollen right thalamus with central hyperdensity indicative of intraparenchymal hemorrhage and associated intraventricular blood within the lateral, third, and fourth ventricles. No midline shift, hydrocephalus, or subarachnoid hemorrhage was observed CT: computed tomography

Cranial ultrasound showed a large area of hyperechogenicity with a thin echogenic capsule in the right thalamus measuring 2.7 × 2.5 × 2.4 cm and spreading into the lateral ventricles; bilaterally, echoic lesions were seen in the caudothalamic grooves, larger on the right, compatible with germinal matrix hemorrhage. These are concerning for hemorrhagic infarction. Follow-up noncontrast computed tomography of the brain showed a swollen hypodense right thalamus with focal intralesional hyperdensity consistent with hemorrhage, along with intraventricular extension to the lateral, third, and fourth ventricles. There was no sign of midline shift, subarachnoid bleeding, or hydrocephalus. The clinical findings were suggestive of right thalamic hemorrhage with secondary IVH (Figure 1).

An in-house pediatric neurosurgeon was unavailable, so the patient was transferred to a tertiary neurosurgical center within 24 hours. She was clinically stable, conscious, and afebrile at admission. She exhibited appropriate limb movements and spontaneous movements, with an age-appropriate tone. The series of head circumference measurements was continuously within the normal range. At the referral hospital, a neurological examination was normal. Pediatric neurology advised starting oral levetiracetam for seizure prophylaxis. There was no recorded seizure at the hospital. The result of the neurosurgical evaluation was that there was no need for emergency surgery because there were no signs of increased intracranial pressure, hydrocephalus, or deteriorating neurological symptoms. A second ultrasound before discharge demonstrated partial improvement in the hemorrhage, no shift of the midline, no additional hemorrhage, and stable ventricles.

She was started on a thorough thrombophilia workup. Laboratory studies were significant for an increased platelet function analyzer (PFA)-100 epinephrine closure time of 228 seconds (reference range 100-180 seconds), suggesting a potential platelet function disorder. The collagen/ADP ratio was normal. The prothrombin time (PT), activated partial thromboplastin time (aPTT), fibrinogen, and D-dimer levels were all normal (Table 1).

**Table 1:** Hematologic and coagulation workup Hematologic, coagulation, and thrombophilia investigations were performed. Routine coagulation parameters were within normal limits, except for a prolonged epinephrine-induced closure time on platelet function analysis PFA-100: platelet function analyzer; FEU: fibrinogen equivalent units

Test	Result	Reference range
Hemoglobin (g/dL)	11.5	10.5–14.0
Platelet count	380 × 10⁹/L	150-450 × 10⁹/L
Prothrombin time (seconds)	13	11-15
Activated partial thromboplastin time (seconds)	32	25-35
Fibrinogen (g/L)	2.8	1.5-4
D-dimer (mg/L FEU)	0.3	<0.5
Antithrombin III (%)	105	80-120
Protein C (%)	85	70-130
Protein S (%)	92	60-150
Factor V Leiden mutation	Negative	-
Homocysteine (µmol/L)	7.2	5-15
PFA-100 (epinephrine) (seconds)	228	100-180
PFA-100 (ADP)	108	60-120

**Figure 2:**
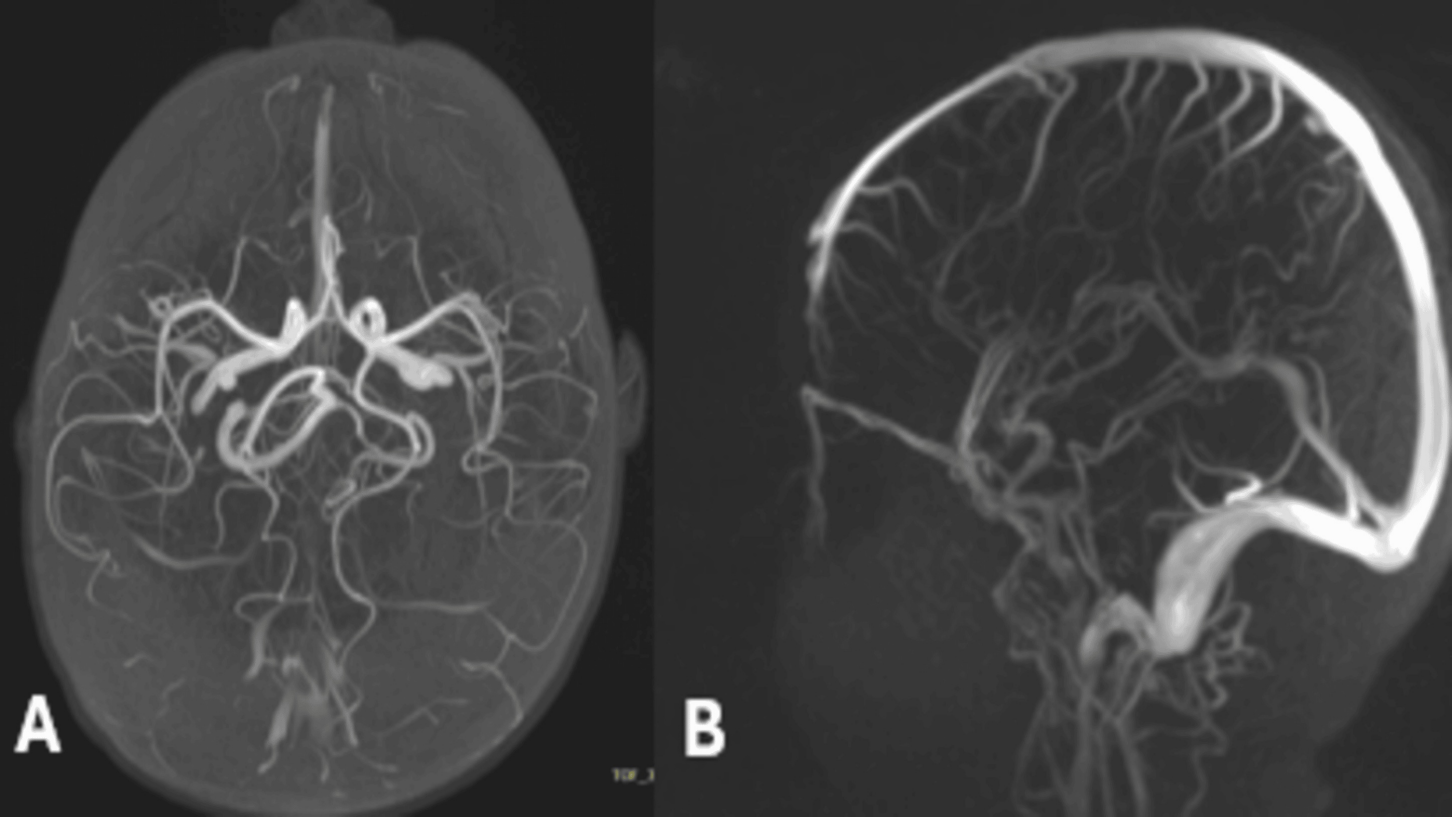
MRA maximum intensity projection images demonstrating normal intracranial arterial anatomy (A) Axial view and (B) sagittal view projection, with preserved anterior and posterior circulation and no evidence of arteriovenous malformation, aneurysm, or arterial stenosis MRA: magnetic resonance angiography

Inherited thrombophilia screening was performed, which showed normal levels of protein C, protein S, and antithrombin III, along with a negative factor V Leiden mutation. Homocysteine levels were normal. Genetic testing for suspected platelet function disorders and thrombophilia was referred to a reference laboratory and did not show genetic mutations. Neuroimaging, including magnetic resonance angiography (MRA), showed that there was no AVM or CVST. The hemorrhage was managed conservatively (Figure 2).

The infant was admitted and managed with an amino acid-based formula for CMPA on supportive prescription care (seizure prophylaxis and nutritional follow-up). She remained clinically stable and was discharged home on oral levetiracetam and vitamin D, with scheduled follow-up appointments at the pediatric neurology, neurosurgery, hematology, and gastroenterology clinics.

At the six-month follow-up, the baby had normal growth and development. She sat with support, exhibited a social smile, maintained visual fixation, and fully recovered age-appropriate neurological function. There were no neurocutaneous stigmas, and the neurological examination was unremarkable (cranial nerves intact, no hydrocephalus, normal tone, and reflexes). She had stopped levetiracetam at the age of four months, with no history of seizures. Follow-up neuroimaging demonstrated resolution of the thalamic hemorrhage and intraventricular blood and no hydrocephalus or mass effect. She continues to be followed up in the neurology and hematology clinics for long-term surveillance.

## Discussion

Spontaneous infantile ICH is an unusual but devastating neurologic emergency that requires both early recognition and a detailed multidisciplinary approach with exclusion of significant underlying etiology. Germinal matrix and IVH in the newborn infant are most often attributed to extreme prematurity and perinatal problems. Spontaneous deep parenchymal hemorrhage in preterm or term infants, except for the neonatal period, is, however, extremely rare, especially in deep brain structures such as the thalamus [1,2].

The thalamus serves as a crucial relay station in the brain. It receives a dense vascular supply from small penetrating arteries that originate from the posterior cerebral and posterior communicating arteries. Its involvement in the setting of hemorrhagic stroke in children is relatively uncommon. In the pediatric population, the most common etiologies of thalamic hemorrhage are AVMs, cavernous malformations, venous sinus thrombosis, hemorrhagic conversion of ischemic infarcts, and coagulopathies [3]. In our case, MRI, MRA, and magnetic resonance venography did not reveal vascular malformations or venous thrombosis, which helped rule out these significant structural etiologies. Moreover, there was no history of trauma, birth asphyxia, or infection, and laboratory data demonstrated that PT, aPTT, fibrinogen, D-dimer, and antithrombin III levels were all within the normal range, so we assumed a lower likelihood of an acquired coagulopathy.

In this case, an inherited thrombophilic disorder was among the essential considerations. Family history was positive for premature venous thrombosis in a maternal aunt who was on long-term anticoagulation, suggesting an inherited prothrombotic disorder. The prolonged epinephrine-induced closure time on the PFA-100 indicated abnormal platelet function and, even if not specific, prompted further genetic analysis. Some hereditary thrombophilias, including protein C or S deficiency, factor V Leiden mutation, prothrombin gene mutation, and platelet adhesion disorders (Glanzmann thrombasthenia, Bernard-Soulier syndrome), can present with cerebrovascular events during infancy, especially in the absence of traditional risk factors [4]. Although no firm diagnosis was established during the index admission, this case demonstrates the value of a thorough thrombophilia workup in the setting of idiopathic hemorrhagic stroke in an infant.

The IVH in this baby is likely a secondary extension from the primary thalamic bleed cited in our previous report, which often accompanies deeper parenchymal bleeds. The occurrence of IVH in the term infant in the absence of trauma or intraventricular instrumentation is an uncommon event. It is usually associated with a more severe clinical course, frequently complicated by hydrocephalus or long-term neurodevelopmental outcome [3]. Of interest is the fact that our patient did not manifest any signs of acute hydrocephalus or intracranial hypertension, and she remained neurologically and developmentally stable during admission and on follow-up. Continuous neuroimaging showed progressive resolution of the hemorrhage and no signs of midline shift or cerebrospinal fluid flow obstruction, so surgical decompression was not necessary.

The clinical diagnosis in this case was initially obscured by the cross-reactive pattern of symptoms in CMPA (a common pediatric disorder affecting 2%-3% of infants) [5]. CMPA frequently manifests with vomiting, diarrhea, irritability, and poor feeding, nonspecific symptoms that could be in significant part confused with an early neurologic presentation. In the present case, gastrointestinal symptoms and recent formula changes prompted initial suspicion of a recurrence of CMPA exacerbations that caused a delay in neurologic evaluation. Persistent upward gaze, lack of ocular pursuit, and high-pitched crying became critical red flags that finally led to urgent neuroimaging. This delay exposes a significant diagnostic pitfall: in infants, one should not interpret behavioral and gastrointestinal symptoms uncritically, especially in the case of deviation from the child's baseline neurological behavior. A very high index of suspicion is required because they present with acute irritability and a loss of developmental milestones, and an alternative diagnosis may seem plausible.

The treatment of spontaneous ICH in the infants is primarily supportive and includes seizure prophylaxis, treatment of increased intracranial pressure, and neurologic monitoring [6]. Surgery, such as ventricular drainage or hematoma evacuation, may be necessary in some instances, especially in the case of hydrocephalus or mass effect [7,8]. Our patient was treated conservatively with oral levetiracetam and closely followed up, which suggests nonoperative treatment in stable patients without progressive hydrocephalus. Long-term follow-up revealed normal neurologic development, recovery of visual tracking, and resolution of the hemorrhage on repeat imaging.

This case highlights key lessons. First, spontaneous deep parenchymal hemorrhage in infancy, though rare, should be considered in infants with neurological changes, even with comorbidities like CMPA [6]. Second, early neuroimaging is crucial with red flag symptoms like abnormal eye movements or altered consciousness [8]. Third, if structural lesions are ruled out, testing for thrombophilia and platelet function is vital, especially with a family history. Finally, conservative management can be suitable with careful follow-up and multidisciplinary input.

## Conclusions

Thalamic hemorrhage occurs spontaneously but is rare in infancy; its nonspecific clinical picture makes diagnosis challenging. This case highlights the importance of maintaining a high degree of suspicion and obtaining early neuroimaging when red flags for neurological signs emerge. A comprehensive work-up for thrombophilia should be considered, particularly when there is a pertinent family history. Early diagnosis and conservative treatment can lead to good neurological outcomes.
